# The Student's Manual of Venereal Diseases

**Published:** 1883-07

**Authors:** 


*The Student's Manual of Venereal Diseases.* By BERKELEY HILL and ARTHUR COOPER. Third Edition, pp. 109. London: Smith, Elder & Co., 1883.

This is an admirable manual, well arranged, clearly written, and free from controversial matter. It contains all that a student need know on the subjects of which it treats, and deserves to be, as it has been, very popular.

